# Correlates of Work‐Related Disability Among Injured Garment Workers in Bangladesh: A Cross‐Sectional Investigation

**DOI:** 10.1002/hsr2.73184

**Published:** 2026-09-01

**Authors:** Md. Golam Kibria, Mohammad Delwer Hossain Hawlader, Mohammad Hayatun Nabi, S. M. Ali Hasnain Fatme, Shakil Ahmed, Mohammad Monjurul Habib, Sheikh M. Alif, Ahmed Hossain

**Affiliations:** ^1^ Department of Research Centre for Development Action Dhaka Bangladesh; ^2^ Department of Public Health North South University Dhaka Bangladesh; ^3^ Department of Program Centre for Disability in Development Dhaka Bangladesh; ^4^ Department of Obstetrics and Gynecology McMaster University Hamilton Ontario Canada; ^5^ Health Department International Federation of Red Cross and Red Cresent Societies Damascus Syria; ^6^ Institute of Health and Wellbeing Federation University Australia Ballarat Australia; ^7^ Health Services Administrations, College of Health Sciences University of Sharjah Sharjah UAE

**Keywords:** Bangladesh, disability, garment worker, RMG industry, workplace injury

## Abstract

**Background and Aims:**

The ready‐made garment (RMG) industry in Bangladesh is crucial for its economy, employing millions of workers. Despite its economic significance, this industry is characterised by unsafe working conditions, resulting in a substantial burden of work‐related injuries. Many injured workers return to their jobs, often experiencing long‐term or permanent disabilities. This study aimed to identify the correlates of work‐related disability among injured garment workers in Bangladesh.

**Methods:**

A cross‐sectional study was conducted among 730 garment workers who had returned to work (RTW) following workplace injuries. Respondents were recruited from 120 conveniently selected RMG factories in Bangladesh. Work‐related disability was assessed using the Washington Group Short Set on Functioning (WG‐SS) questions. Multivariable logistic regression was performed to identify the factors associated with work‐related disability.

**Results:**

The prevalence of work‐related disability among injured workers was 13%, with the majority (58.9%) experiencing mobility disability. Male workers (adjusted odds ratio, aOR: 2.0; 95% confidence interval, CI: 1.24 to 3.18), those aged < 30 years (aOR: 2.9; 95% CI: 1.06 to 8.18), and workers from the cutting section (aOR: 2.2, 95% CI: 1.42 to 3.32) had higher odds of work‐related disability. Injury‐related factors associated with a higher likelihood of work‐related disability included bone fractures (aOR: 3.7; 95% CI: 1.81 to 7.53), burn injuries (aOR: 2.4; 95% CI: 1.38 to 4.18), same‐level slips or trips (aOR: 3.8; 95% CI: 2.99 to 4.72), and fire accidents (aOR: 8.2; 95% CI: 1.44 to 46.11).

**Conclusions:**

This study identified sociodemographic, occupational, and injury‐specific correlates of work‐related disability among injured RMG workers in Bangladesh. These findings highlight the need for interventions to prevent work‐related disability and support injured workers. They also provide evidence to inform occupational health policies and future research.

## 1 Background

Work‐related disability is a common public health issue in the ready‐made garment (RMG) industry of low‐ and middle‐income countries (LMICs), including Bangladesh. According to the World Health Organization (WHO), disability is an umbrella term, covering impairments, activity limitations, and participation restrictions [1]. Work‐related disability is a type of disability that results from occupational injuries or illnesses caused by prolonged exposure to workplace hazards [2]. In the RMG industry, common work‐related injuries encompass both traumatic events, such as cuts, lacerations, punctures, dislocations, fractures, and burns and musculoskeletal disorders (MSDs), including pain in the back, neck, shoulders, ankles, knees, and wrists, and hands [3, 4, 5, 6]. These traumatic events usually result from physical and chemical hazards, including machinery accidents, sharp objects, and toxic substances. On the other hand, poor ergonomics, repetitive tasks, prolonged standing, and heavy lifting are significant contributors to MSDs [7, 8]. The prevalence of occupational injuries among garment workers in LMICs is alarmingly high. For instance, the 1‐year prevalence of occupational injuries in the garment industry has been reported at 27.8% in Ethiopia and 35.3% in Sri Lanka [9, 10]. In Bangladesh, the RMG industry saw 241 fire‐related incidents in a single year, 2022 [11]. Moreover, 24.7% of garment workers experienced acute traumatic events, such as cuts and abrasions, during the previous year [12], and 57% reported musculoskeletal pain in at least one body part during the last month [6].

Work‐related disabilities have physical, economic, and psychological impacts on affected persons, their families, and employers. Persons with severe injuries or disabilities experience immobility, poor dexterity, and low muscular strength, which limit their ability to perform occupational and daily activities [13, 14]. Existing evidence shows that employers are often reluctant to recruit or retain workers with disabilities due to concerns about reduced productivity and expenses related to disability pensions, workplace modifications, and accommodations [15, 16]. Even when persons with disabilities retain their employment, they usually receive lower wages and occupational demotions [17, 18]. Managing disabilities is a long‐term process that imposes significant out‐of‐pocket expenses on disabled workers and their families for consultations, medications, rehabilitation, assistive devices, and extended care [19, 20, 21]. Furthermore, disabilities contribute to increased levels of anxiety, depression, stress, post‐traumatic stress disorder (PTSD), and, in severe cases, suicidality [22, 23, 24, 25]. A study conducted by the Centers for Disease Control and Prevention (CDC), USA, has found that adults with disabilities are 4.6 times more likely to experience frequent mental distress compared to those without disabilities [26].

Bangladesh, with a population of over 165 million, depends on the RMG industry as a key driver of its economy. The sector is the country's largest employer, which employs 4.1 million workers, over 60.0% of whom are women [27]. In the fiscal year 2022‐2023, it contributed USD 47.38 billion to exports, representing 84.6% of national export earnings and positioned Bangladesh as the world's second‐largest RMG exporter [28, 29]. Despite its substantial economic contribution, the RMG industry continues to face persistent occupational safety challenges, including machinery accidents, fire hazards, structural failures, chemical exposures, inadequate lighting, heavy lifting, and insufficient use of personal protective equipment (PPE) [30, 31, 32]. These hazards collectively increase the risk of both acute traumatic injuries and chronic musculoskeletal conditions, thereby amplifying the likelihood of work‐related disabilities.

To date, there is limited empirical evidence regarding the prevalence and correlates of work‐related disability among garment workers in Bangladesh. This knowledge gap poses a serious barrier to developing evidence‐based interventions and policies that could prevent injuries, reduce disability, and enhance worker safety and well‐being. Addressing this gap is critical given the economic significance of the RMG sector and its reliance on a predominantly young workforce. Against this background, the present study aimed to examine the factors associated with work‐related disability among injured garment workers in Bangladesh.

## 2 Methodology

### 2.1 Study Design and Participants

This cross‐sectional study was conducted under the *Employment Injury Protection Scheme for Workers in the Textile and Leather Industries (EIPS)* project (https://www.giz.de/en/projects/employment-injury-protection-scheme-workers-textile-and-leather-industries). This study design was chosen because it enables the estimation of the prevalence of work‐related disability and the examination of associations between disability and potential demographic, occupational, and injury‐related factors within a defined population at a single point in time. The study included garment workers who (a) were aged 18 years or older, (b) had been continuously employed in garment factories for at least 1 year to the injury, and (c) had sustained a work‐related injury within the 5‐year period from 1 January 2014 to 31 March 2019. Garment workers who were employed in factories outside Dhaka and Gazipur districts, had retired, or were unable to communicate were excluded. A work‐related injury was defined as any traumatic event (e.g., cuts, fractures, burns, dislocations) or musculoskeletal disorder (e.g., pain in the back, neck, or limbs) arising from occupational hazards or workplace activities.

### 2.2 Study Setting and Sampling

There are a total of 4,134 garment factories in Bangladesh, of which over 80% are concentrated in four districts – Dhaka, Gazipur, Narayanganj, and Chattogram [33]. For this study, Dhaka and Gazipur districts were selected as study sites because together they accommodate more than half of the country's garment factories and represent the principal garment manufacturing hubs. Selecting these districts maximised the likelihood of recruiting a sufficiently large and diverse sample of injured garment workers while remaining operationally feasible. Dhaka is located in the geographic centre of the country, while Gazipur is a neighbouring district situated about 40 kilometres to the north of Dhaka city.

**FIGURE 1:**
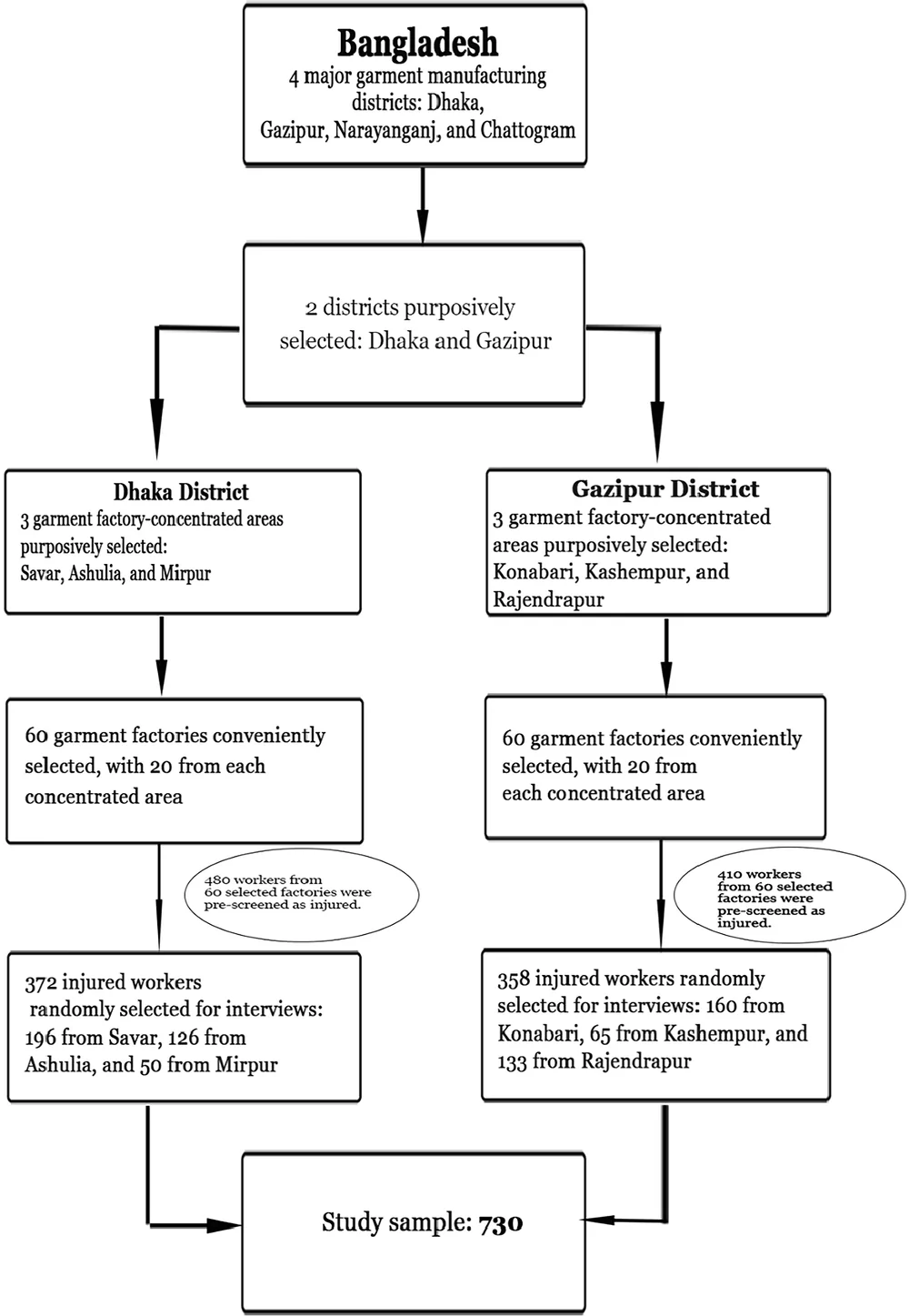
Multistage cluster sampling procedure used to select respondents for the study.

A multistage cluster sampling technique was used to recruit injured garment workers from major garment manufacturing areas in Bangladesh. The required sample size was calculated using the following formula, *n* = $\frac{\left(z_{\alpha /2}\right)2p(1-p)}{e^{2}}$ [34], where *n* is the desired sample size, *z* is the standard normal deviate (1.96 at 95% confidence interval), *p* is the estimated prevalence of work‐related disability among garment workers who had experienced workplace injuries (50%), and e is the margin of error (5%). As no previous study had estimated the prevalence of work‐related disability among injured garment workers in Bangladesh, a prevalence of 50% was assumed to maximise the sample size and ensure adequate statistical precision. Based on this calculation, the initial sample size was 384. After adjusting for a design effect of 1.8 to account for clustering within garment factories and a 5% non‐response rate, the minimum sample size for this study was 728. In total, 750 eligible respondents were approached for interviews, of whom 730 eventually participated. The sampling procedure comprised four stages (Figure 1). In the first stage, out of the four major garment manufacturing districts, Dhaka and Gazipur districts were purposively selected for this study. In the second stage, three garment factory‐concentrated areas were purposively chosen from each district: Savar, Ashulia, and Mirpur from Dhaka, and Konabari, Kashempur, and Rajendrapur from Gazipur. In the third stage, 120 garment factories (20 from each selected area) were selected using convenience sampling because access depended on obtaining permission from factory management and logistical feasibility during the study period. In the final stage, garment workers were screened using a single question to determine whether they had experienced a workplace accident or injury during the past 5 years. Workers who answered “yes” were then asked brief follow‐up questions regarding the time, place, and cause of the injury to confirm its work‐related nature. From the resulting sampling frame of 890 injured workers, 730 were randomly selected for interviews, including 372 from Dhaka and 358 from Gazipur.

### 2.3 Measures

#### 2.3.1 Outcome Variable

Respondents' work‐related disability was measured using the Washington Group Short Set on Functioning (WG‐SS) questions [35]. The WG‐SS consists of six questions on core functional domains: vision, hearing, mobility, cognition, self‐care, and communication. Each question is rated on a four‐point Likert scale: 1 = no difficulty, 2 = some difficulty, 3 = many difficulties, and 4 = cannot do it at all. Respondents who agreed with either category 3 or 4 to at least one of the six questions were classified as having a disability. The Cronbach's alpha value of the WG‐SS used in the present study was 0.61, indicating that the scale had modest internal reliability.

#### 2.3.2 Independent Variables

##### 2.3.2.1 Sociodemographic Characteristics

Sociodemographic variables used in this study were sex (male or female), age (< 30 years, 30 to 45 years, or > 45 years), educational attainment (no education/primary, secondary, or higher secondary), marital status (unmarried or ever married), number of earning household members (1 persons, 2 persons, or > 2 persons), and monthly household income (< 20,000 Bangladeshi Taka‐ BDT, 20,000 to 30,000 BDT, or > 30,000 BDT). Household income is the total combined income from all sources received by all individuals residing in a single household.

##### 2.3.2.2 Occupational Characteristics

Occupational factors included job tenure (< 3 years, 3 to 7 years, or > 7 years), department/section (sewing, cutting, dyeing & printing, finishing, or administration), position (machine operator, machine operator assistant/helper, general worker, supervisor/floor‐in‐charge, cleaner/loader, iron man/laundry man, or quality checker), daily working hours (8 h or > 8 h), and compensation (full, partial, or no). In this study, daily working hours were defined as the total number of hours a worker spends at the workplace each day, encompassing both standard working hours (8 h) and overtime. Participants were asked whether they received any financial compensation for days lost due to work‐related injuries.

##### 2.3.2.3 Clinical and Injury‐Related Characteristics

Clinical and injury‐related factors used in this study were diabetes mellitus (yes, no, or do not know), cardiovascular disease (yes, no, or do not know), respiratory disease (yes, no, or do not know), mental illness (yes, no, or do not know), type of injury (cut/abrasion/laceration, needlestick injury, musculoskeletal disorder, bone fracture, burn injury, eye/ear injury, burn injury, or dislocation), source of injury (machinery, slip/trip, poor ergonomics, environmental exposure, collision with objects, or fire accidents), and hospitalisation (admitted or non‐admitted). In this study, *cardiovascular diseases* included hypertension, heart disease, and stroke; *respiratory diseases* comprised asthma, chronic obstructive pulmonary disease, and tuberculosis (TB); and *mental illness*es encompassed anxiety, depression, stress, and insomnia. Participants were asked whether they had ever been diagnosed with or currently experienced any of these conditions through a structured questionnaire, with response options of yes, no, or do not know.

### 2.4 Data Collection

Four teams, each consisting of two data collectors and one field supervisor, were recruited for data collection. Each team member held at least a bachelor's degree in social sciences and had prior experience in data collection. Before fieldwork, all team members received 2 days of training covering the survey questionnaire, interview techniques, and ethical issues. Following the training, a semi‐structured survey questionnaire, comprising three sections (a) sociodemographic characteristics, (b) occupational information, and (c) clinical factors, was pretested among 20 injured RMG workers to determine its clarity, practicality, and relevance to the study. The questionnaire was originally developed in English, then forward‐translated into Bengali and backward‐translated into English to ensure linguistic and conceptual consistency. Based on pretest feedback, necessary revisions were made, and the instrument was finalised for data collection.

Respondents were recruited after obtaining written permission from the authorities of the selected garment factories. On scheduled dates, data collectors conducted face‐to‐face interviews in the factories during office hours, only after written informed consent was secured from each respondent. On prescheduled dates, data collectors conducted face‐to‐face interviews using the survey questionnaire within the factories during office hours only after written informed consent was secured from each respondent. Privacy was maintained by conducting interviews in private and semi‐private areas to prevent responses from being overheard by other workers or supervisors. Respondents were assured that all information would remain confidential and be used solely for research purposes. Data collection was conducted over a two‐and‐a‐half‐month period from June to August 2019. Each interview lasted approximately 30 min, and each respondent received 200 BDT as a token of appreciation. Quality control measures included spot checks by field supervisors to verify the completeness and clarity of both coded and open‐ended responses. Additionally, the principal investigator and other key researchers periodically monitored data collection to ensure consistency and quality.

### 2.5 Statistical Analysis

Statistical analyses were performed using Stata version 17. Three variables had missing data: age, monthly household income, and job tenure, and the percentages of missingness at the item level ranged from 0.7% to 1.8%. Little's MCAR test with *χ*2 = 9.011, *p* = 0.173 indicated that the data were completely missing at random [36]. Missing values were handled using the expectation maximisation (EM) method. However, frequencies and percentages were used to describe the sociodemographic, occupational, and clinical and injury‐related characteristics of respondents. Bivariate associations between work‐related disability and selected categorical variables were assessed using the chi‐square test of independence. Fisher's exact test was used for those categorical variables that had expected frequency less than five observations in any cell [37]. Binary logistic regression (LR) was used to identify the factors associated with work‐related disability. Independent variables were selected for inclusion in the LR model based on a combination of statistical significance in the bivariable analyses at *p* < 0.05 and a priori knowledge. Independent variables that were found to be significant in the bivariable analyses at *p* < 0.05 were sex, age, educational attainment, department/section, type of injury, source of injury, site of injury, and hospitalisation. However, the goodness‐of‐fit of the LR model was assessed using the Hosmer‐Lemeshow test. Multicollinearity was evaluated using the variance inflation factor (VIF); all independent variables had VIF values below 5, with a mean VIF of 2.39, indicating no significant multicollinearity [38]. Due to multicollinearity, the site of injury was excluded from the LR model. Although job tenure was not significant in the bivariable analysis, it has been identified as a significant factor for work‐related injuries in the earlier literature [39, 40]. Therefore, the final LR model was adjusted for sex, age, educational attainment, department/section, type of injury, source of injury, and job tenure. To account for clustering at the garment factory‐concentrated area level, standard errors were estimated using Huber's robust variance estimator in Stata [41]. The results of the LR analyses were presented as adjusted odds ratios (aORs) with 95% confidence intervals (CIs). All statistical tests were two‐sided, and *p*‐values < 0.05 were considered statistically significant.

## 3 Results

### 3.1 Sociodemographic Characteristics

**FIGURE 2:**
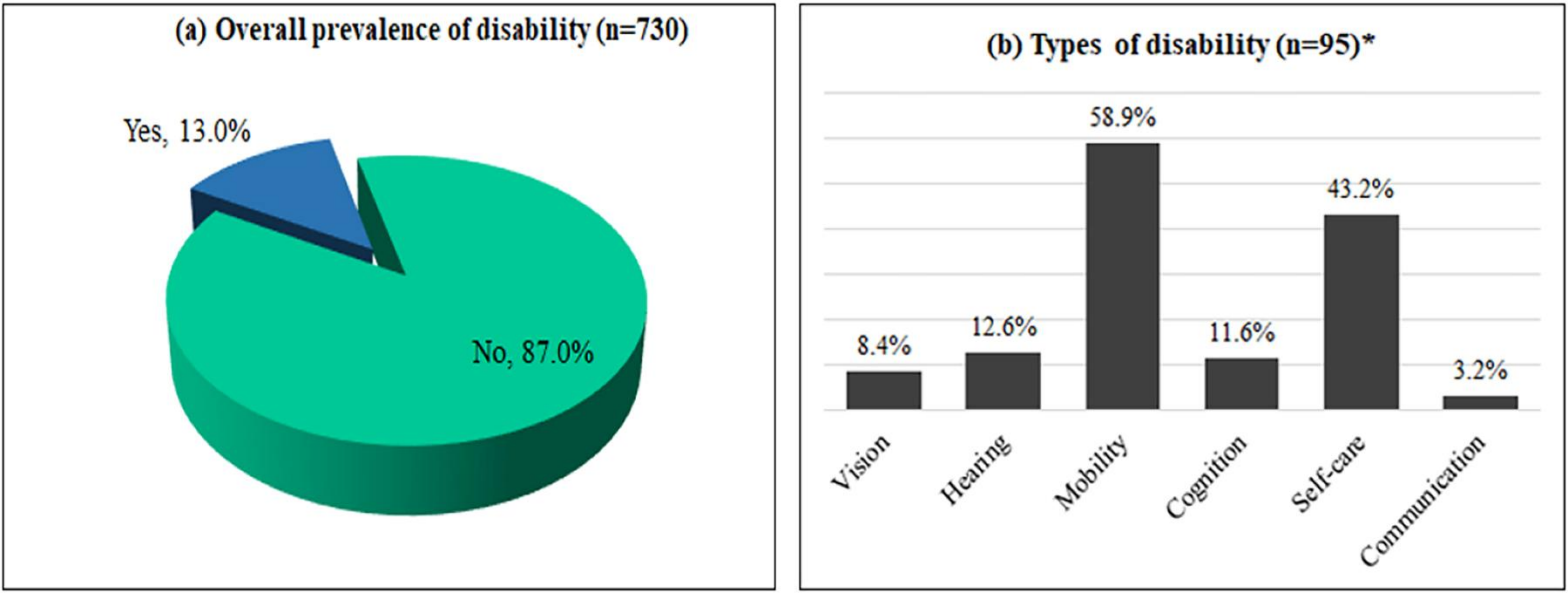
Prevalence and types of work‐related disability among injured garment workers. *Percentages were calculated using multiple response analysis.

Figure 2 presents the overall prevalence and types of work‐related disability among injured garment workers. Of the 730 study respondents, 13% reported experiencing at least one disability. Regarding the types of disability, the most common was mobility disability (58.9%), followed by self‐care disability (43.2%).

Table 1 presents the distribution of the study sample according to sociodemographic characteristics and their bivariable associations with work‐related disability. The majority of respondents were male (53.3%), under 30 years of age (50.4%), completed secondary education (52.5%), were ever married (78.2%), and reported two earning members in the household (61.2%). Approximately half of the respondents (49.2%) had a monthly household income of 20,000 to 30,000 Bangladeshi Taka (BDT).

**TABLE 1:** Distribution of respondents according to sociodemographic factors and their bivariable associations with work‐related disability.

Variable		Disability		*p* value^a^
	Total (*n* = 730)	Present (*n* = 95)	Absent (*n* = 635)	
	*n* (%)	*n* (%)	*n* (%)	
Sex				
Female	341 (46.7)	35 (10.3)	306 (89.7)	**0.039**
Male	389 (53.3)	60 (15.4)	329 (84.6)	
Age				
< 30 years	368 (50.4)	59 (16.0)	309 (84.0)	**0.041**
30 to 45 years	253 (34.7)	27 (10.7)	226 (89.3)	
> 45 years	109 (14.9)	9 (8.3)	100 (91.7)	
Educational attainment				
Higher secondary	56 (7.7)	7 (12.5)	49 (87.5)	**0.037**
Secondary	383 (52.5)	39 (10.2)	344 (89.8)	
No education/Primary	291 (39.9)	49 (16.8)	242 (83.2)	
Marital status				
Unmarried	159 (21.8)	20 (12.6)	139 (87.4)	0.854
Ever married	571 (78.2)	75 (13.1)	496 (86.9)	
Number of earning members				
1 person	193 (26.4)	24 (12.4)	169 (87.6)	0.392
2 persons	447 (61.2)	63 (14.1)	384 (85.9)	
> 2 persons	90 (12.3)	8 (8.9)	82 (91.1)	
Monthly household income				
< 20,000 BDT	318 (43.6)	48 (15.5)	261 (84.5)	0.184
20,000 to 30,000 BDT	359 (49.2)	42 (11.6)	321 (88.4)	
> 30,000 BDT	53 (7.3)	5 (8.6)	53 (91.4)	

*Note:* Bold values indicate significant results.

^a^ Pearson's chi‐square test.

The bivariable analysis results show that work‐related disability was significantly associated with several sociodemographic factors, including sex (*p* = 0.039), age (*p* = 0.041), and educational attainment (*p* = 0.037).

### 3.2 Occupational Characteristics

Table 2 illustrates the distribution of study respondents according to occupational factors and their bivariable associations with disability. Most respondents worked in the garment industry for less than 3 years (47.5%), were employed in the sewing section (54.9%), held positions as machine operators (54.4%), and reported working > 8 h per day (70.4%). Furthermore, over 40% of respondents were provided financial compensation for lost workdays due to workplace injuries, of which 32.3% were fully compensated and 9.6% were partially compensated.

**TABLE 2:** Distribution of respondents according to occupational characteristics and their bivariable associations with work‐related disability.

Variable		Disability		*p* value^a^
	Total (*n* = 730)	Present (*n* = 95)	Absent (*n* = 635)	
	*n* (%)	*n* (%)	n (%)	
Job tenure				
< 3 years	347 (47.5)	53 (15.3)	294 (84.7)	0.085
3 to 7 years	252 (34.5)	32 (12.7)	220 (87.3)	
> 7 years	131 (17.9)	10 (7.6)	121 (92.4)	
Department/Section				
Sewing	401 (54.9)	42 (10.5)	359 (89.5)	**0.008**
Cutting	130 (17.8)	19 (14.6)	111 (85.4)	
Dyeing & Printing	48 (6.6)	14 (29.2)	34 (70.4)	
Finishing	110 (15.1)	15 (13.6)	95 (86.4)	
Administration	41 (5.6)	5 (12.2)	36 (87.8)	
Position				
Machine Operator	397 (54.4)	53 (13.4)	344 (86.6)	0.404^b^
Machine Operator Assistant/Helper	110 (15.1)	18 (16.4)	92 (83.6)	
General worker	79 (10.8)	4 (5.1)	75 (94.9)	
Supervisor/Floor‐in‐Charge	57 (7.8)	7 (12.3)	50 (87.7)	
Cleaner/Loader	45 (6.2)	7 (15.6)	38 (84.4)	
Iron Man/Laundry Man	25 (3.4)	3 (12.0)	22 (88.0)	
Quality Checker	17 (2.3)	3 (17.6)	14 (82.4)	
Daily working hours				
8 h	216 (29.6)	36 (16.7)	180 (83.3)	0.057
> 8 h	514 (70.4)	59 (11.5)	455 (88.5)	
Compensation				
Full	236 (32.3)	202 (85.6)	34 (14.4)	0.090
Partial	70 (9.6)	56 (80.0)	14 (20.0)	
No	424 (58.1)	377 (88.9)	47 (11.1)	

*Note:* Bold values indicate significant results.

^a^ Pearson's chi‐square test.

^b^ Fisher's exact test.

The bivariable analysis results indicate a statistically significant association between work‐related disability and only one occupational factor, department/section (*p* = 0.008).

### 3.3 Clinical and Injury‐Related Characteristics

Table 3 presents the distribution of respondents according to clinical and injury‐related characteristics and their bivariable associations with work‐related disability. A substantial proportion of injured garment workers suffered from diabetes mellitus (11.2%), cardiovascular diseases (8.6%), respiratory diseases (13.6%), and mental illnesses (25.2%). Approximately one‐fourth of respondents experienced MSDs (29.9%), followed by cuts, abrasions, or lacerations (23.2%). The majority of respondents reported machinery as the source of their workplace injuries (55.9%) and the upper limbs as the site of injuries (54%). A notable proportion of respondents (14.8%) were hospitalised following workplace injuries.

**TABLE 3:** Distribution of respondents according to clinical characteristics and their bivariable association with work‐related disability.

Variable		Disability		*p* value^e^
	Total (*n* = 730)	Present (*n* = 95)	Absent (*n* = 635)	
	*n* (%)	*n* (%)	*n* (%)	
Diabetes mellitus				
Yes	82 (11.2)	14 (17.1)	68 (82.9)	0.405
No	479 (65.6)	72 (12.9)	488 (87.1)	
Do not know	169 (23.2)	9 (10.2)	79 (89.8)	
Cardiovascular disease				
Yes	63 (8.6)	8 (12.7)	55 (87.3)	0.604
No	438 (60.0)	53 (12.1)	385 (87.9)	
Do not know	229 (31.4)	34 (14.8)	195 (85.2)	
Respiratory disease				
Yes	99 (13.6)	9 (9.1)	90 (90.9)	0.395
No	484 (66.3)	64 (13.2)	420 (86.8)	
Do not know	147 (20.1)	22 (15.0)	125 (85.0)	
Mental illness				
Yes	184 (25.2)	24 (13.0)	160 (87.0)	0.551
No	369 (50.5)	52 (14.1)	317 (85.9)	
Do not know	177 (24.2)	19 (10.7)	158 (89.3)	
Type of injury				
Cut/Abrasion/Laceration	169 (23.2)	12 (7.1)	168 (92.3)	**< 0.001** f
Needlestick injury	157 (21.5)	9 (5.7)	148 (94.3)	
aMusculoskeletal disorder	218 (29.9)	27 (12.4)	191 (87.2)	
Bone fracture	56 (7.7)	22 (39.3)	34 (60.7)	
Blunt injury	45 (6.2)	6 (13.3)	39 (86.7)	
Eye/ear injury	41 (5.6)	4 (9.8)	37 (90.2)	
Burn injury	25 (3.4)	8 (32.0)	17 (68.0)	
Dislocation	19 (2.6)	7 (36.8)	12 (63.2)	
Source of injury				
bMachinery	408 (55.9)	26 (6.4)	382 (93.6)	**< 0.001**
Slip/Trip	129 (17.7)	39 (30.2)	90 (69.8)	
cPoor ergonomics	94 (12.9)	10 (10.6)	84 (89.4)	
dEnvironmental exposure	55 (7.5)	6 (10.9)	49 (89.1)	
Collision with objects	29 (4.0)	6 (20.7)	23 (79.3)	
Fire accident	15 (2.1)	8 (53.3)	7 (46.7)	
Site of injury				
Head/Face/Neck	67 (9.2)	8 (11.9)	59 (88.1)	**0.028**
Upper limb	394 (54.0)	40 (10.2)	354 (89.8)	
Trunk	107 (14.7)	22 (20.6)	85 (79.4)	
Lower limb	162 (22.2)	25 (15.4)	137 (84.6)	
Hospitalisation				
Admitted	108 (14.8)	31 (28.7)	77 (71.3)	**< 0.001**
Non‐admitted	622 (85.2)	64 (10.3)	558 (89.7)	

*Note:* Bold values indicate significant results.

^a^ Musculoskeletal disorders included foot pain, ankle pain, knee pain, thigh pain, low back pain, hip pain, joint pain, upper back pain, shoulder pain, neck pain, chest pain, wrist pain, sprain, and back injury in this study.

^b^ Sewing machines, cutting machines, automated equipment, scissors, knives, and shears.

^c^ Awkward working posture, lifting heavy objects, repetitive movement, and long working hours.

^d^ Exposure to chemicals, noise, heat, and poor light.

^e^ Pearson's chi‐square test.

^f^ Fisher's exact test.

The bivariable analysis results show that work‐related disability was significantly associated with several clinical and injury factors, including type of injury (*p* < 0.001), source of injury (*p* < 0.001), site of injury (*p* = 0.028), and hospitalisation (*p* < 0.001).

### 3.4 Factors Associated With Work‐Related Disability

Table 4 presents the results of multivariable logistic regression analysis examining the correlates of work‐related disability. Injured male workers were 2.0 times more likely to develop work‐related disability compared to their female counterparts (95% CI: 1.24 to 3.18). Respondents aged below 30 years had 2.9 times higher odds of experiencing disability compared to those aged above 45 years (95% CI: 1.06 to 8.18). According to the department/section category, workers from the cutting section were at 2.2 times increased risk of work‐related disability compared to those from the sewing section (95% CI: 1.42 to 3.32). Garment workers who sustained bone fractures in the workplace were 3.7 times more likely to develop disability compared to those who experienced cuts, abrasions, or lacerations (95% CI: 1.81 to 7.53). Respondents with burn injuries had 2.4 times higher odds of experiencing work‐related disability compared to those with cuts, abrasions, or lacerations (95% CI: 1.38 to 4.18). Respondents who had injuries from same‐level slips or trips were at 3.8 times higher risk of developing work‐related disability compared to those who were injured while operating machinery (95% CI: 2.99 to 4.72). Similarly, garment workers who had injuries from fire accidents demonstrated an 8.2‐fold increased risk of disability compared to those injured during machine operation (95% CI: 1.44 to 46.11).

**TABLE 4:** Multivariable binary logistic regression analysis to investigate correlates of work‐related disability (*n* = 730).

Variable	aOR	95% CI	*p* value
Sex			
Female	Reference		0.004**
Male	2.0	1.24 to 3.18	
Age			
> 45 years	Reference		0.038*
30 to 45 years	1.9	0.88 to 4.09	
< 30 years	2.9	1.06 to 8.18	
Department/Section			
Sewing	Reference		
Cutting	2.2	1.42 to 3.32	< 0.001***
Dyeing & Printing	2.3	0.46 to 11.29	0.313
Finishing	0.9	0.18 to 4.46	0.899
Administration	0.9	0.14 to 5.74	0.910
Type of injury			
Cut/Abrasion/Laceration		Reference	
Needlestick injury	1.4	0.48 to 3.89	0.557
aMusculoskeletal disorder	1.7	0.47 to 6.15	0.414
Bone fracture	3.7	1.81 to 7.53	< 0.001***
Eye/Ear injury	1.6	0.32 to 7.91	0.563
Blunt injury	1.4	0.99 to 1.93	0.052
Burn injury	2.4	1.38 to 4.18	0.002**
Dislocation	4.3	0.73 to 25.45	0.106
Source of injury			
Machinery	Reference		
Slip/Trip	3.8	2.99 to 4.72	< 0.001***
Poor ergonomics	1.1	0.62 to 2.10	0.683
Environmental exposure	1.1	0.11 to 11.08	0.947
Collision with objects	2.9	0.84 to 9.67	0.092
Fire accident	8.2	1.44 to 46.11	0.017*

Abbreviations: aOR, adjusted odds ratio; CI, confidence interval.

^a^ Musculoskeletal disorders included foot pain, ankle pain, knee pain, thigh pain, low back pain, hip pain, joint pain, upper back pain, shoulder pain, neck pain, chest pain, wrist pain, sprain, and back injury in this study.

* *p*‐value < 0.05

** *p*‐value < 0.01

*** *p*‐value < 0.001.

## 4 Discussion

This study identified several key factors associated with work‐related disability among injured RMG workers in Bangladesh. The findings of this study can inform the development of more adaptable policies and strategies aimed at safeguarding the occupational health and safety of manufacturing workers, particularly those in the RMG sector.

This study revealed that 13% of injured garment workers in Bangladesh experienced at least one form of disability. This prevalence is slightly higher than the estimated prevalence of disability in the general population of Bangladesh (9.1%) [42]. This elevated prevalence could be attributed to the greater vulnerability of garment workers to injuries and disability, resulting from poor workplace ergonomics, exposure to hazardous chemicals and machinery, lack of training, and limited access to timely medical care. To address this, factory authorities should strengthen compliance with safety standards, as outlined in the ILO Convention on Occupational Safety and Health [43], the Bangladesh Labour (Amendment) Act 2013 [44], and the National Occupational Safety and Health Policy 2013 [45]. Recommended measures include conducting regular inspections, providing safety training, ensuring consistent use of PPE, and establishing transparent mechanisms for injury reporting and timely compensation. Additionally, access to physiotherapy, vocational retraining, and psychological support should be ensured to promote recovery and facilitate successful reintegration into the workforce.

Mobility disability was identified as the most common type of disability in the present study, which is consistent with earlier research [46, 47]. This finding underscores the need to prioritise interventions targeting mobility limitations among injured garment workers. Factories should implement ergonomic workplace improvements, including redesigned workstations and adjustable seating to reduce unnecessary movements. Structured rehabilitation programmes should be offered, such as on‐site physiotherapy, early intervention, and adaptive job assignments, should be offered to support recovery and maintain mobility. Furthermore, workers should have access to mobility aids when needed, and supervisors should monitor for early signs of mobility impairment.

The multivariable analysis of this study revealed that male workers were at greater risk of developing work‐related disability compared to their female counterparts. This finding is supported by the results reported in another Bangladeshi study [48]. This increased risk among men could be attributed to their greater engagement in physically demanding or high‐risk tasks, which are associated with more severe injuries. However, other previous studies have reported no inherent sex differences in the risk of work‐related injury or disability; instead, differences in risk are largely determined by the type and nature of work performed across sexes [49, 50]. Such findings suggest that sex alone may not be a causal factor, and that occupational exposures, task‐specific hazards, and workplace conditions could play a critical role in shaping disability risk. To mitigate these risks, factories should monitor task allocation to minimise excessive exposure to high‐risk tasks among male workers and implement sex‐sensitive training programmes. Future research should examine the interaction between sex, task type, and workplace environment to better understand underlying mechanisms.

The findings of this study indicate that respondents aged below 30 years had higher odds of experiencing work‐related disability compared to those aged above 45 years. This finding is similar to those of previous studies reporting that younger workers are more susceptible to occupational injuries, which may lead to work‐related disabilities [51, 52]. However, this finding should be interpreted with caution because the number of disability cases among younger workers was relatively small, which may have reduced the precision and stability of the estimated association. The observed association may be explained by the fact that younger workers are more often assigned to physically demanding or high‐risk tasks, such as cutting, lifting heavy materials, or operating machinery in the garment or manufacturing industry, where their limited experience and lower awareness of occupational hazards increase vulnerability [53]. Another possible explanation is that younger workers may have less familiarity with workplace safety protocols, further increasing their susceptibility to occupational injuries and subsequent disability [54]. Nevertheless, further studies with larger and more representative samples are needed to confirm the relationship between age and work‐related disability among RMG workers.

According to the findings of the present study, garment workers in the cutting section are more likely to experience work‐related disability compared to those in the sewing section. This elevated risk could be explained by the inherent nature of cutting tasks, which involve the frequent use of sharp tools and heavy cutting machinery, and repetitive fabric‐handling activities. Such exposures increase the likelihood of acute traumatic injuries such as deep lacerations, amputations, as well as long‐term musculoskeletal disorders from repetitive lifting and awkward postures, often leading to permanent functional impairment and disability [55, 56]. In contrast, sewing work, while physically demanding and commonly associated with musculoskeletal disorders of the neck, back, shoulders, hands, and wrists [57], generally involves repetitive yet low‐intensity tasks that tend to result in more gradual and less severe health outcomes, which may not necessarily progress to permanent disability. These findings underscore the need for targeted preventive strategies in the cutting section, including comprehensive safety training, use of appropriate PPE, ergonomic interventions, task rotation, and regular occupational health monitoring to reduce the risk of injuries and long‐term disability among garment workers.

The present study identified bone fractures and burn injuries as a significant risk factor for work‐related disability. Garment workers who sustained bone fractures in the RMG industry were more likely to develop disabilities compared to those who experienced cuts, abrasions, or lacerations. Bone fractures are typically more severe injuries that often require prolonged immobilisation, surgical intervention, or extensive rehabilitation, leading to reduced mobility, decreased muscle strength, and limitations in performing repetitive or physically demanding tasks [58, 59, 60, 61, 62]. Respondents who suffered from burn injuries in the workplace were at higher risk of disability compared to those who had cuts, abrasions, or lacerations. Workplace burns may result from thermal, chemical, electrical, or radiation hazards [63]. Burn injuries can result in long‐term physical effects, including scarring, contractures, and chronic pain, as well as psychological consequences such as anxiety, depression, and PTSD, which can limit daily and occupational functioning [64, 65, 66]. To mitigate the risk of both bone fractures and burn injuries, garment factories should implement comprehensive safety measures, including strict enforcement of standard operating procedures, provision and mandatory use of appropriate PPE, regular hazard assessments, and routine maintenance of machinery and electrical systems. Workers should receive targeted training on safe material handling, machinery operation, and burn prevention. Structured rehabilitation programmes and psychological support should also be available to support optimal recovery.

In this study, two sources of injury‐ slips or trips and fire accidents were found to be significantly associated with work‐related disability. Garment workers who experienced injuries due to same‐level slips or trips were at higher risk of developing work‐related disability. This association is consistent with findings from previous studies, which have shown that slips or trips are a major contributor to occupational injuries and subsequent disability in industrial settings [67, 68]. Slips generally result from low‐friction or slippery surfaces, such as wet, oily, or spill‐covered floors, whereas trips occur when an individual encounters obstacles along their walking path, including clutter, uneven surfaces, objects on the floor, or poor lighting [69, 70]. To prevent slips and trips, it is crucial to implement measures, such as maintaining clean and dry floors, installing anti‐slip surfaces, keeping walkways unobstructed, and ensuring adequate workplace lighting. This study also revealed that respondents who experienced injuries from fire accidents in the workplace had higher odds of developing disability. This finding aligns with prior evidence, showing that burn injuries are among the most severe occupational hazards, often resulting in deep tissue damage, contractures, amputations, and disfigurement, which significantly impair physical functionality [71, 72]. To mitigate the risk and impact of fire‐related injuries, garment factory management should strengthen fire safety infrastructure, including alarms, sprinklers, and emergency exits; ensure regular inspection of electrical systems to prevent short circuits; provide fire safety training and evacuation drills; and supply flame‐resistant protective equipment in high‐risk areas.

Although this study identified several physical and occupational correlates of work‐related disability, psychosocial factors, including mental health, workplace culture, and workplace and social support, may also influence disability outcomes and return‐to‐work among injured workers. These factors were beyond the scope of the present study but warrant further investigation to provide a more comprehensive understanding of work‐related disability in the RMG sector.

### 4.1 Policy Implications

The high prevalence of work‐related disability observed in this study highlights the need for comprehensive occupational safety and rehabilitation strategies within Bangladesh's RMG sector. The World Health Organization (WHO) and the International Labour Organization (ILO) recommend workplace interventions, including hazard identification, ergonomic improvements, worker safety training, appropriate use of PPE, and effective occupational safety and health (OSH) management systems to prevent occupational injuries and promote worker safety [73, 74, 75, 76]. Furthermore, systematic reviews have shown that coordinated return‐to‐work programmes involving healthcare providers, employers, and workers can improve functional recovery, facilitate work participation, and reduce work disability [77, 78]. These recommendations are consistent with the ILO Guidelines on Occupational Safety and Health Management Systems (ILO‐OSH 2001), which emphasise hazard prevention, risk management, worker participation, and continuous improvement of workplace safety [74]. In Bangladesh, strengthening the implementation of occupational safety regulations, improving compliance with OSH standards, expanding access to occupational rehabilitation services, and integrating early rehabilitation into routine injury management may help reduce the burden of work‐related disability and improve functional recovery among injured garment workers.

### 4.2 Strengths and Limitations

A major strength of this study is its comparatively large, multi‐factory sample, which enhances the representativeness of the study population. Another strength of the study is the use of multivariable logistic regression, which allows for the simultaneous adjustment of potential confounders, thereby providing more reliable estimates of the associations between work‐related disability and sociodemographic, occupational, and clinical and injury‐related factors.

Several limitations should be considered when interpreting the findings. First, this study used a cross‐sectional design that cannot establish a causal relationship [34]. Second, the use of self‐reported data may have introduced recall and social desirability bias. Third, convenience sampling from selected RMG factories may have introduced selection bias and limited the generalisability of the findings. Future studies should consider probability‐based sampling across a larger and more geographically diverse sample of factories to improve the representativeness and external validity of the findings. Fourth, less common injury types were not analysed separately because of their low frequency, which limited the statistical power to estimate reliable associations. Fifth, although the WG‐SS is widely used internationally, it has not been formally validated in the Bangladeshi population. Therefore, caution is warranted when interpreting disability estimates derived from this instrument. Sixth, this study included only injured garment workers who had returned to work but excluded those unable to resume work due to a severe work‐related injury or disability. This exclusion may have resulted in an underestimation of the prevalence and severity of work‐related disability. Finally, although this study included a wide range of variables, several potentially important factors, including comorbidities, health‐seeking behaviour, access to rehabilitation, psychological support, workplace support, and organisational culture, were not measured. In addition, the absence of qualitative data limited the exploration of injured workers' lived experiences, perceptions, and coping strategies. These unmeasured factors may have influenced the observed associations and limited a comprehensive understanding of the correlates and broader consequences of work‐related disability. Future studies should adopt longitudinal mixed‐methods designs that incorporate these factors to provide more robust and comprehensive evidence.

## 5 Conclusions

This study identified several factors associated with work‐related disability among injured garment workers in Bangladesh, including younger age, male gender, employment in the cutting section, shorter job tenure, bone fractures, burn injuries, slips or trips, and fire accidents. These findings underscore the need for interventions to prevent work‐related disability and support injured workers in the RMG sector. They also provide evidence to inform occupational health policies and prioritise workers at greater risk of disability. Further research is needed to evaluate the effectiveness of interventions aimed at reducing work‐related disability and improving recovery among injured garment workers.

## Author Contributions

**Md Golam Kibria:** conceptualization, data curation, investigation, validation; formal analysis, funding acquisition, visualization, project administration, writing – original draft, writing – review and editing. **Mohammad Delwer Hossain Hawlader:** conceptualization, methodology, investigation, validation, funding acquisition, writing – review and editing. **Mohammad Hayatun Nabi:** conceptualization, methodology, investigation, validation, funding acquisition, writing – review and editing. **S. M. Ali Hasnain Fatme:** investigation, project administration, writing – review and editing. **Shakil Ahmed:** data curation, investigation, project administration, writing – review and editing. **Mohammad Monjurul Habib:** methodology, investigation, project administration, writing – review and editing. **Sheikh M. Alif:** validation, visualization, writing – review and editing. **Ahmed Hossain:** conceptualization, methodology, data curation, investigation, validation, supervision, funding acquisition, writing – review and editing.

## Ethics Statement

The study protocol was approved by the National Ethics Review Committee (NERC) of the Bangladesh Medical Research Council (Ethics approval number: 20808052019). The study was conducted in accordance with the ethical standards and regulations of the ethics committee.

## Conflicts of Interest

The authors declare no conflicts of interest.

## Transparency Statement

Md. Golam Kibria affirms that this manuscript provides an honest, accurate, and transparent account of the study being reported, with no important aspects omitted. All authors have had full access to the data presented, approved the final version for publication, and any discrepancies from the study as originally planned have been fully accounted for and explained.

## Data Availability

The data that support the findings of this study are available on request from the corresponding author. The data are not publicly available due to privacy or ethical restrictions.
